# Nutrient addition alters plant community productivity but not the species diversity of a mountain meadow in Tajikistan

**DOI:** 10.3389/fpls.2023.1235388

**Published:** 2024-01-15

**Authors:** Okhonniyozov Mekhrovar, Yao-ming Li, Madaminov Abdullo, Yusupov Sino, Lianlian Fan

**Affiliations:** ^1^ State Key Laboratory of Desert and Oasis Ecology, Xinjiang Institute of Ecology and Geography, Chinese Academy of Sciences, Urumqi, China; ^2^ Research Center for Ecology and Environment of Central Asia, Chinese Academy of Sciences, Urumqi, China; ^3^ Research Center for Ecology and Environment of Central Asia, Dushanbe, Tajikistan; ^4^ University of Chinese Academy of Sciences, Beijing, China; ^5^ Institute of Botany, Physiology and Plant Genetics of the Academy of Sciences of the Republic of Tajikistan, Dushanbe, Tajikistan

**Keywords:** *Prangos pabularia* Lindl., nutrient addition, above-ground biomass, grassland, Central Asia

## Abstract

**Introduction:**

Tajikistan is a typical mountainous country covered by different mountain grasslands that are important pasture resources. Recently, grassland degradation has become widespread due to climate change and human activities and fertilization has been used to improve grassland production. However, fertilizer inputs can substantially alter species diversity, but it is uncl\ear how productivity and species diversity respond to nutrient enrichment in the mountain meadows of Tajikistan.

**Methods:**

Therefore, a 5-year (2018–2022) continuous *in-situ* mineral fertilizer experiment was conducted to examine the effects of three nitrogen (N) levels (0, 30, and 90 kg N ha^–1^ year^–1^), two phosphorus (P) levels (0 and 30 kg P ha^–1^ year^–1^), and their combinations on above-ground biomass (AGB) and species diversity in a mountain meadow grassland in Ziddi, Varzob region, Tajikistan. Five species diversity metrics—Margalef’s species richness (D_ma_), the Shannon–Wiener index (H), the Simpson index (C), Pielou’s equitability index (E_pi_), and the Evar Species Evenness index (E_var_)—were used to measure species diversity.

**Results and discussions:**

The results indicated that the addition of different N and P amounts and their various combinations considerably increased both total and dominant species AGB, with the highest increase occurring in the N90P30 (90 kg N ha^–1^ year^–1^ combined with 30 kg P ha^–1^ year^–1^) treatment in 2022; during the experiment, the importance value of *Prangos pabularia* (dominant species) first decreased and then increased, but its dominant status did not change or fluctuate among the years. Furthermore, N, P, and their different combinations had no significant effect on species diversity (D_ma_, H, C, E_pi_, and E_var_). All the species diversity indexes fluctuated among years, but there was no interaction with mineral fertilizer addition. Total AGB had a negative relationship with species diversity and low concentration N fertilizer addition (N30; P30) strengthened this negative trend. However, this trend decreased under the high N fertilizer condition (N90P30). Overall, nutrient addition to the natural mountain grassland of the Varzob region improved AGB, which meant that there was more forage for local animals. Mineral fertilizers had no significant effect on species diversity, but may enhance *P. pabularia* dominance in the future, which will help maintain the stability of the plant community and improve the quality of the forage because *P. pabularia* is an excellent and important winter fodder. Our study suggests that scientific nutrient management could effectively promote grassland production, conserve plant variety, and regenerate degraded grassland, which will counteract the desertification process in northwest Tajikistan mountain meadows.

## 1 Introduction

Almost 30% of the global land surface is covered by grassland, which means that it is an important terrestrial ecosystem (Bi et al., 2020). The production of feed for livestock and the preservation of biodiversity are just two of the numerous ecological services that grassland ecosystems provide. Grassland ecosystems have undergone significant changes in recent decades as a result of climate change and human activities, both of which have triggered dynamic fluctuations in these ecosystems (Jiang et al., 2015; Su et al., 2021). The fundamental characteristics of grassland structure and function include plant productivity and species richness, which may partially affect system stability (Wu et al., 2020). As a consequence of climate change, changes in these characteristics have become difficult to predict and the degradation of grasslands, which is influenced by both human activity and climate change, is becoming more serious.

The application of fertilizer to grassland soils may impact yields, soil nutrients, and their availability (Qin et al., 2015). Organic fertilizers, such as animal dung, muck crops, compost, crop wastes, and sewage sludge, were vital components of integrated nutrient management systems in traditional farming because they enabled the soil to retain its beneficial characteristics (Frossard et al., 2009). However, as modern agriculture has advanced, mineral-based fertilizers, such as nitrogen and phosphorus fertilizers, have replaced organic fertilizers. Nutrient addition can impact the structure and variety of grassland plant communities and this can affect grassland productivity. Studies on grassland plant community composition, structure, and variety in response to nutrient addition have crucial theoretical and practical value for the scientific management of grassland, the protection of plant diversity, and the recovery of damaged grassland (Yu et al., 2015; Li et al., 2018). Nitrogen (N) and phosphorus (P) are the two most limiting elements affecting grassland productivity and N is generally considered to be the predominant limiting nutrient (Ma et al., 2008; Robertson and Vitousek, 2009). Many fertilizer studies have shown that the availability of mineral nutrients, particularly N, is a major factor affecting grassland productivity (Guo et al., 2010). In many ecosystems, aboveground biomass (AGB) increases when N is added (Liu et al., 2014). Another important mineral component for crop production is P, which enhances soil fertility and maintains the soil mineral equilibrium (Selim et al., 2009; Demirsoy et al., 2012; Memon et al., 2012; Hartley et al., 2013). In many grasslands, nitrogen and phosphorus are among the most significant limiting elements because of their critical roles in plant development (Fay et al., 2015). Worldwide, grassland systems have been shown to benefit greatly from fertilization in terms of increasing productivity (Ineichen et al., 2020).

Tajikistan is a typical mountainous country in Central Asia with considerable amounts of grassland. The grassland distribution mainly extends between 300 and 4000 m above sea level, resulting in various microclimates and grassland varieties (Fan et al., 2021). Grasslands are crucial resources for the Tajikistan economy. However, due to the rapid growth in population and the increased need for food and meat, overgrazing and other usage practices have led to a significant degradation in grasslands over the last three decades (Han et al., 2016). The degradation of grasslands and desertification are the two main issues affecting the ecosystem and resources of northwest Tajikistan. Therefore, it is important to investigate methods that can be used to increase forage biomass without affecting species biodiversity (Muminjanov, 2008). Previous studies showed that the application of fertilizer, mainly N and P, enhances productivity in different types of grasslands in Tajikistan by 2.5–3 times, but it is accompanied by a change in their floristic composition and structure (height, vertical distribution of the mass of aboveground organs, number of tillers, etc.) (Madaminov, 2000).

In this study a 5-year (2018–2022) *in-situ* mineral addition experiment was conducted in Ziddi, Varzob district, northwest Tajikistan, where the local mountain meadow grassland has become degraded due to overuse and a lack of adequate management. The dominant species in the study area is *Prangos pabularia* Lindl, a perennial herbaceous plant that contributes almost half of the grassland biomass. Therefore, the effects of fertilizer (N, P) addition on AGB and species diversity were studied. The hypotheses were that N and P fertilizers and their combinations increased the biomass of aboveground plants and improved biodiversity (Botter et al., 2020; Bedaso et al., 2021; Pavlina et al., 2015), especially *P. pabularia* biomass, and that nutrient addition would enhance *P. pabularia* dominance as it is an excellent fodder crop.

## 2 Materials and methods

### 2.1 Study area description

**Figure 1:**
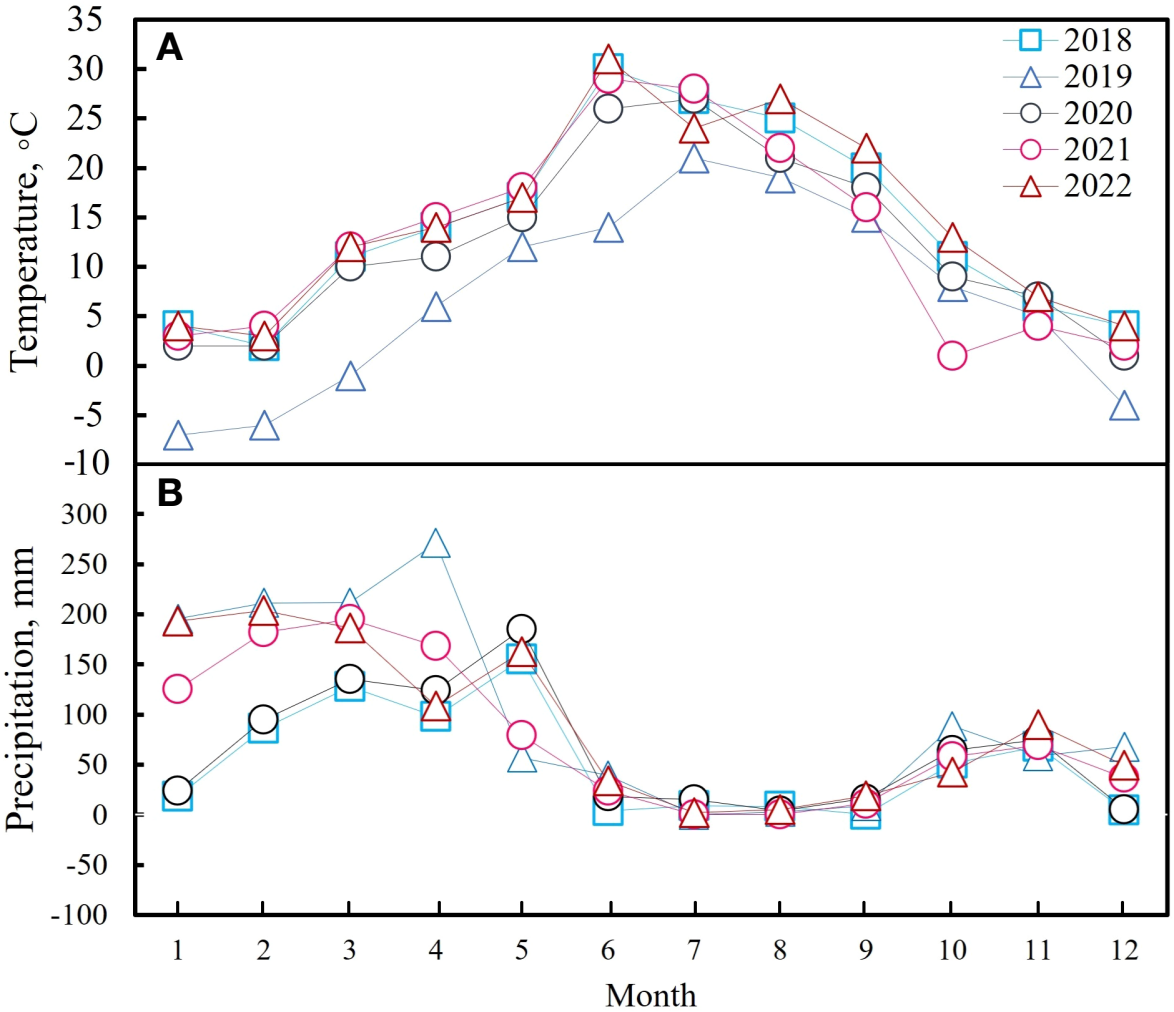
The average monthly temperatures **(A)** and total precipitation **(B)** for each of the five years.

The experiment was carried out between 2018 and 2022 on the southern slope of the Hissar ridge in the lower part of the watershed for the Ziddy River, northwest Tajikistan, latitude 39°02.190°N, longitude 68°49.370°E, altitude 2000 m. The annual average temperature is 12.2°C (**Figure 1A**) and the annual mean rainfall is 942 mm, which mainly falls between December and April (**Figure 1B**). This area is classified as a mountain meadow grassland. The main soil in the experimental field is Cambisol (typical brown) under the World Reference Base soil classification system. It is derived from the parent rock, contains gravelly-stony limestone, and the upper horizon contains 3.5%–5.0% humus*. Prangos pabularia* Lindl is the dominant species in this grassland, but it also contains regional plants, mainly pasture plants, such as *Geranium collinum* Stephan ex Willd, *Vicia tenuifolia* Roth, *Lathyrus inconspicuous* L., *Astragalus peduncularis* Benth, *Medicago sativa* L., *Bromus inermis* Leyss, *Dactylis glomerata* L., *Hordeum bulbosum* L., *Taraxacum officinale* F. H. Wigg, *Crepis sibirica* L., *Polygonum coriarium* Grig, *Convolvulus arvensis* L., *Artemisia absinthium* L., *Cousinia umbrosa* Bunge, *Bromus oxyodon* Schrenk, *Elaeosticta hirtula* (Regel & Schmalh.) Kljuykov, Pimenov & V. N. Tikhom, *Erophila verna* L., *Lathyrus pratensis* L., *Plantago lanceolata* L., *Poa bulbosa* L., *Ranunculus laetus* L., *Scabiosa songarica* (Schrenk) Soják, and *Trifolium repens* L.

### 2.2 Experimental design

This study investigated the impact of nutrient addition on pasture ecosystem production and species richness. A 20 × 15 m experimental site was set up in the Ziddi grassland area, which is dominated by *P. pabularia.* The blocks were separated by at least a 1 m wide buffer zone and each block was subsequently subdivided into five plots for the various nutrient treatments. Each plot was 3 × 3 m in size with a 1 m buffer zone between them (**Supplementary Figure 1**). The N was supplied as urea (CH_4_N_2_O), the phosphate was supplied as phosphorus pentoxide (P_2_O_5_), and the treatment without nutrient addition was the control (CK). Each block had five nutrients treatments: CK–the control; N30 - 30 kg N ha^–1^ year^–1^, P30 - 30 kg P ha^–1^ year^–1^, N30P30 - 30 kg N h a^–1^ year^-1^ and 30 kg P ha^–1^ year^–1^, and N90P30 - 90 kg N ha^–1^ year^–1^ and 30 kg P ha^–1^ year^–1^. Each treatment was replicated four times and fertilizer application took place from the 15^th^ May after the snow had melted. To prevent water penetration between treatments, all treatment zones were surrounded by stainless steel sheets that were sunk into the soil for approximately 40 cm with 10 cm left exposed above the ground.

### 2.3 Plant sampling

The number of species (species richness) were counted each year in a 1 × 1 m quadrat (randomly placed) (**Supplementary Figure 1**) under the various fertilizer treatments when growth was at its peak (middle May to early June). The Gult-Drude scale was used to characterize abundance. At the end of the growth period, the AGB in the 1 m × 1 m quadrats was gathered from the surface. The height, phenological phase, and abundance of each plant type were recorded. The height of the herbage and general aspects of phytocenosis were also recorded. All the above ground material from each quadrat was placed in envelopes, dried to a constant weight in an oven at 68°C, and the dry weight was calculated as the AGB.

### 2.4 Data analysis

The following indices were used to assess the plant communities (Magurran, 1998; Whittaker et al., 2001):

Species importance value (IV) (Ismail et al., 2017; Tong et al., 2019)


(1) IV=(RH+RC+RF+RD)/4

where RH is relative height, RC is relative coverage, RF is relative frequency, and RD is relative density.

Species α diversity index (D_ma_) using the Margalef’s species richness index (Margalef, 1973):


(2) $$D_{ma}=\left(\text{S}-1\right)/\ln\text{N}$$

where S is the number of each species and N is the number of all species.

Shannon–Wiener diversity index (H) (Niu et al., 2018):


(3) H=−∑pilnPi

where *Pi* is the proportion of individuals of a given species.

Simpson’s dominance index (C) (Simpson, 1949):


(4) $$\text{C}=1-\sum Pi^{2}$$

where *Pi* is the proportion of individuals of a given species.

Pielou’s equitability index (E_pi_) (Smith and Wilson, 1996):


(5) $$E_{pi}=\text{H}/\ln\text{S}$$

where H is the Shannon–Wiener diversity index and S is the community species numbers.

Species Evenness Index (E_var_)


(6) $$E_{var}=1-\frac{2}{\pi }arctan\left\{\sum_{S=1}^{S}{\left(\ln\left(x_{S}\right)-\sum_{t=1}^{S}\ln\left(x_{1}\right)/S\right)}^{2}/S\right\}$$

where S is the number of species in the sample, χ*_S_* is the abundance of the species, and arctan provides the angle in radians. The variance (Equation 6) was the log of the abundances and was used to examine proportional differences and to ensure the index was not dependent on the units used. The variance was converted by 2/pr arctan() to a 0–1 range, with 0 representing minimum evenness and 1 representing maximum evenness (Smith and Wilson, 1996).

All data were analyzed, and the graphs were created using Microsoft Excel (Redmond, WA, USA) and Origin Pro 2021 (OiginLab Corporation, Northampton, MA, USA). The significance of the treatments, assessed using a linear mixed-effects model, the total ABG, species diversity importance value, and *P. pabularia* biomass (PB), natural height, density, and coverage were all calculated using SPSS statistical software (Chicago, Il, USA). Based on their relevance, a cluster tree of all species in the entire experiment was created using an averaging method. The level of statistical significance was set at 0.05.

## 3 Results

### 3.1 Impact of nutrient addition on above-ground biomass

**Figure 2:**
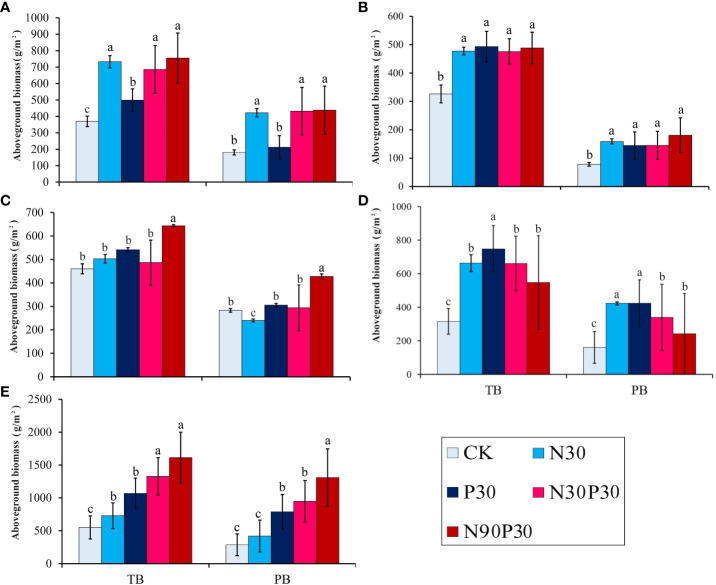
Total above-ground biomass (TB) and *P. pabularia* above-ground biomass (PB) in the different fertilizer treatments from 2018–2022 **(A–E)** (g m^–2^, mean ± SE). Different letters (a–c) indicate significant differences at the *P*< 0.05 level by Duncan’s test.

The major species, *P. pabularia*, was most impacted by the fertilizer applications. **Figure 2** shows the impact of the various treatments on total AGB and PB in the different years. The harvest time varied between years, which meant that comparing the mean biomasses and other indicators was difficult. In 2018, the total AGB in N90P30 considerably increased to 919.20 g·m^−2^ compared to CK (**Supplementary Table 2**) and the PB also significantly increased in all treatments (*P<* 0.05). The PB increased from 78.10 g·m^−2^ (CK) to 241.24 g·m^−2^ (N90P30) in 2019 (*P<* 0.05) and the total AGB increased to 598 g·m^−2^ (*P<* 0.05). The results for 2020 were similar to those for 2019. However, in 2021 and 2022, there were significant changes. In 2021, the total AGB significantly increased to 1440.58 g·m^−2^ in N90P30 (*P<* 0.05) and PB also significantly increased for the different treatments, especially in N90P30. However, the total AGB and PB increased dramatically to 2252.50 g·m^−2^ and 1745.67 g·m^−2^, respectively, for N90P30 in 2022. Interestingly, compared to the other years, the total AGB and PB increased with nutrient addition (**Supplementary Table 2**).

Overall, the continuous mineral fertilizer application treatments had greater total AGBs and *P. pabularia* levels in 2021 and 2022, especially for the N90P30 treatment (*P<* 0.05) (**Figure 2**). In this study, both years and treatments strongly influenced total AGB and PB, but there was no interaction effect (*P* > 0.05) and they independently affected AGB and PB (**Table 1**).

**Table 1:** Summary of the linear mixed-effect model relating fixed factors (Y, T) for grassland total above-ground biomass and *P. pabularia* above-ground biomass.

Source	AGB		PB	
	*F*	*P*	*F*	*P*
Y	12.615	0.000***	10.015	0.000***
T	5.265	0.001	2.801	0.032
Y×T	1.522	0.114	1.312	0.213

Y represents different years, T represents the different treatments, and Y × T is their interaction. ***Significant difference at the P< 0.05 level.

### 3.2 Importance value index for *P. pabularia* over the five years

**Figure 3:**
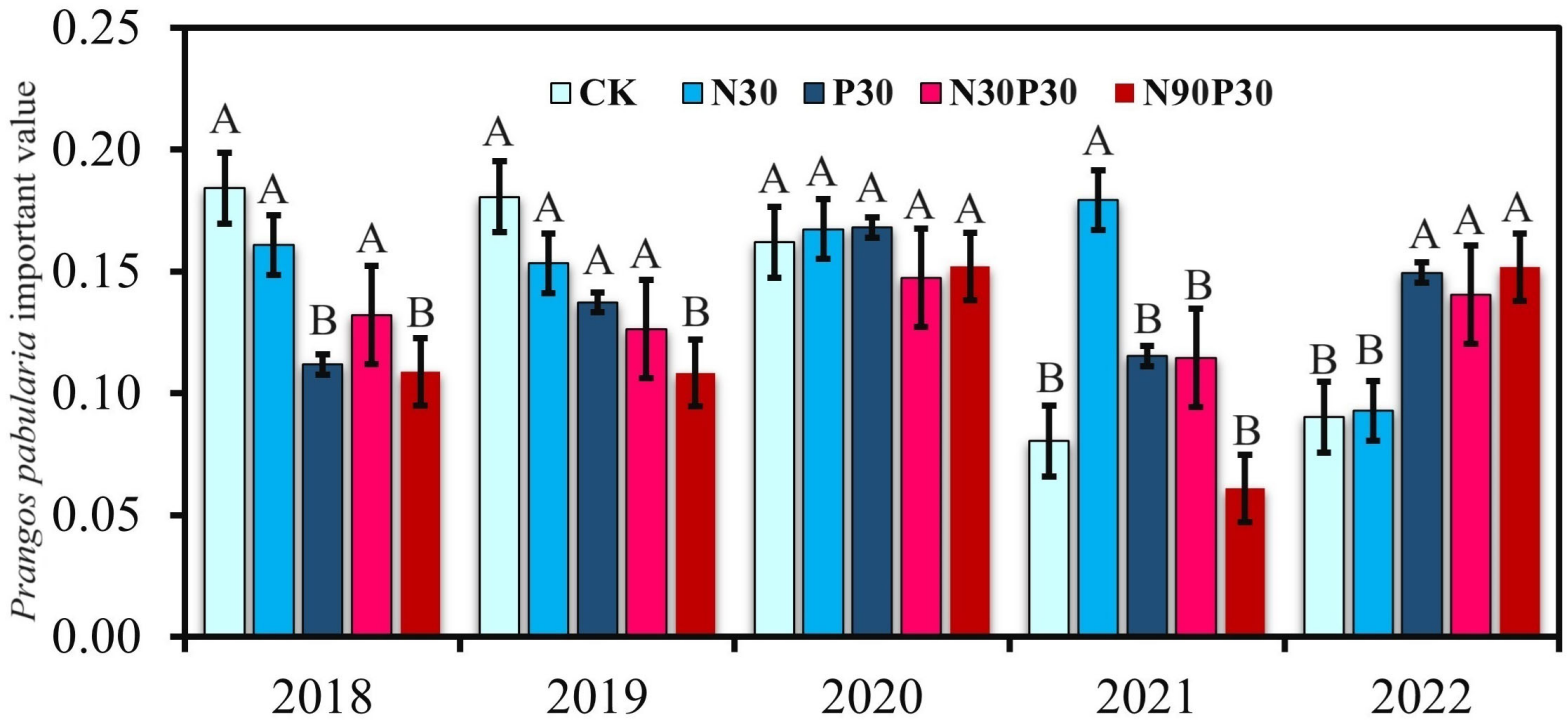
*Prangos pabularia* Lindl importance values. For each year, significant differences (*P*< 0.05) among the treatments are indicated by uppercase letters (A, B). For each treatment, there were no significant differences (ns) among the years.

The importance values (IVs) for *P. pabularia* over the five years are shown in **Figure 3** (using Equation 1). The P30, N30P30, and N90P30 treatments significantly decreased the *P. pabularia* IVs in 2018 and 2019 compared to CK. None of the treatments had a significant effect on IV in 2020 and 2021and only N30 increased the IV. In contrast to 2018 and 2019, the P30, N30P30, and N90P30 treatments significantly increased the IV in 2022 compared to CK. Furthermore, the *P. pabularia* IV also changed with years under the same treatment. The IV for CK did not change from 2018 to 2020 but decreased in 2021 and 2022. The IV decreased for N30 in 2022 and increased for P30 in 2020 and 2022, whereas the N30P30 and N90P30 treatments had no obvious effect on the IV from 2018–2021, but increased the IV in 2022.

**Figure 4:**
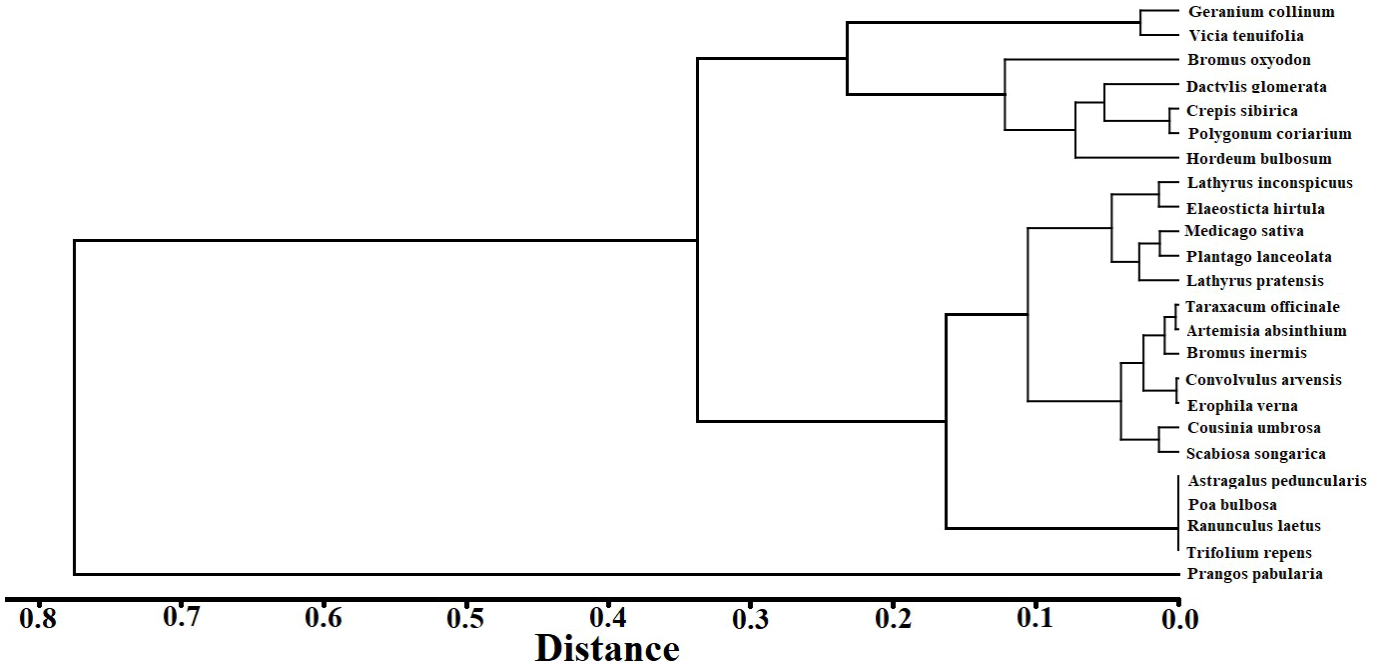
Species cluster tree map of the importance values.

A cluster tree analysis by species IV for the 2020 CK treatment was undertaken (**Figure 4**). Based on the tree map, four species groups were identified between 0.2 and 0.3 spaces. The initial group was *P. pabularia* alone, the next group was *Geranium collinum* and *Vicia tenuifolia*, the third was *Astragalus peduncularis, Poa bulbosa, Ranunculus laetus*, and *Trifolium repens*, and the fourth group contained all of the remaining species, such as *Taraxacum officinale, Crepis sibirica, Polygonum coriarium, Convolvulus arvensis, Artemisia absinthium, Cousinia umbrosa, Bromus oxyodon*, and *Elaeosticta hirtula*.

*Prangos pabularia* and *G. collinum* have evolved naturally over thousands of years to become the most prevalent species in the Ziddi Region. The *P. pabularia* IVs were high for every treatment in each year (**Figure 4**), which showed that it was the most significant independent species in this grassland.

### 3.3 Species proportions within the plant community

**Figure 5:**
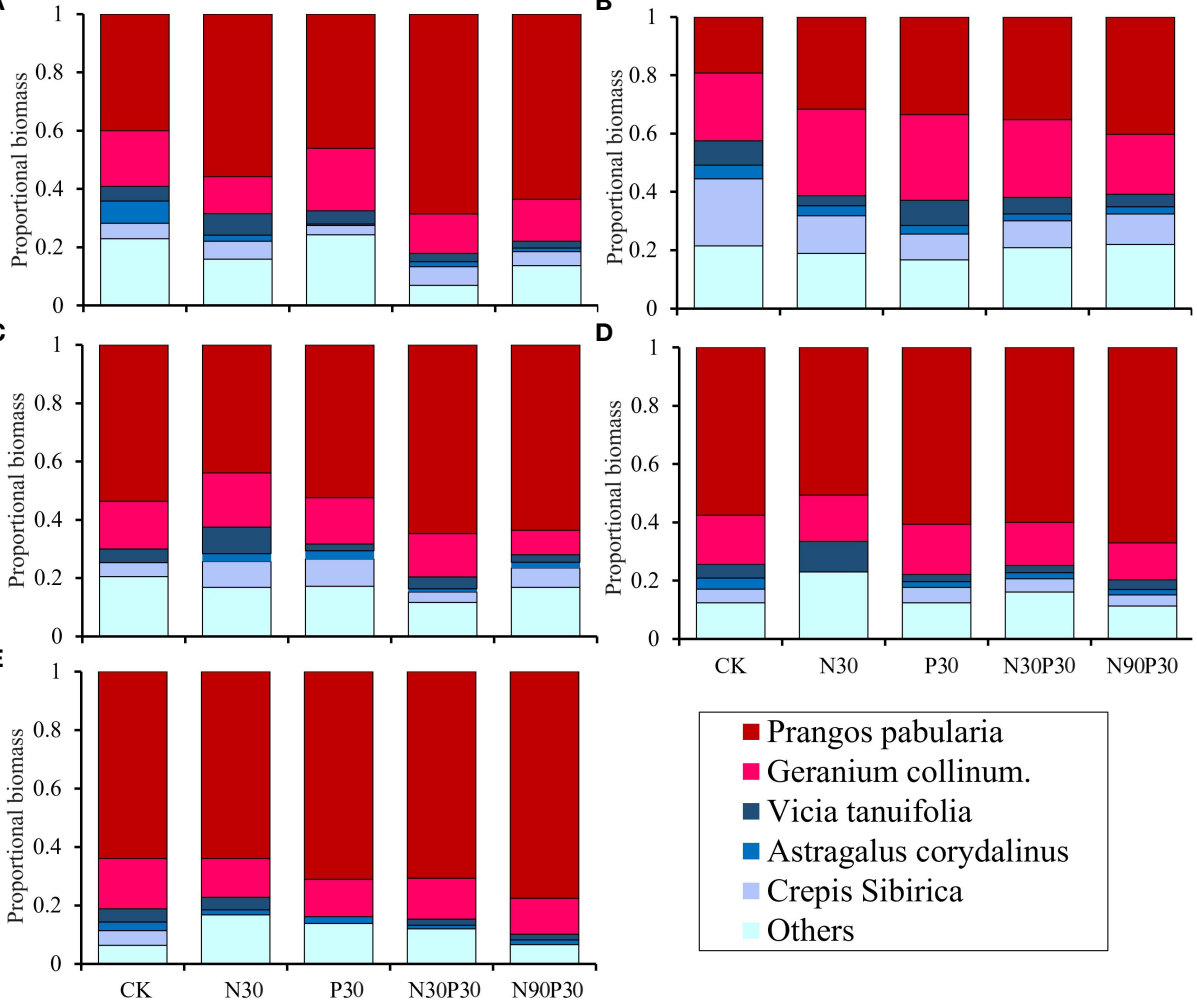
Effects of fertilizers on proportional plant above-ground biomass for the dominant species in the grassland from 2018–2022 **(A-E)**.

Six categories were used to group the plant species biomass proportions: *P. pabularia*, *G. collinum*, *V. tenuifolia*, *A. peduncularis*, *C. sibirica*, and others (**Figure 5**). The results showed that *P. pabularia* biomass was highest in all the treatments, followed by *G. collinum* and others (*P<* 0.05, **Supplementary Table 3**). There was an overall decreasing trend in the biomass proportions of *A. peduncularis* and *C. sibirica* with increasing years of fertilization (*P<* 0.05). Furthermore, from 2018–2022, the N90P30 treatment greatly increased the *P. pabularia* biomass compared to the other treatments. The biomasses of the remaining species varied less between treatments. In general, mineral fertilizer enhanced *P. pabularia* and *G. collinum* biomass production while limiting the growth of *C. sibirica* (**Figure 5**).

### 3.4 Species biodiversity changes

**Figure 6:**
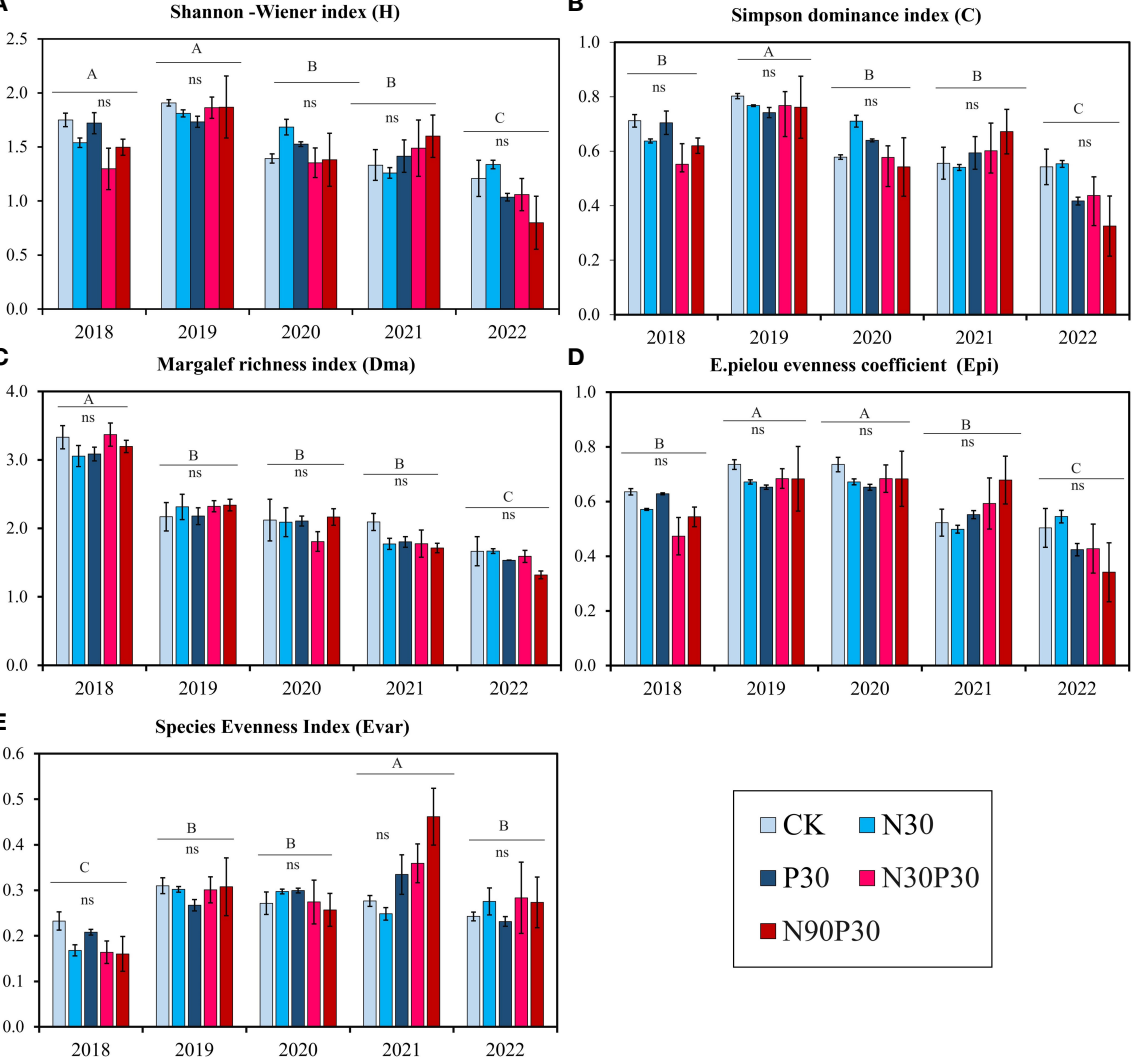
Biodiversity indexes during a five-year period (2018–2022). **(A)** Shannon–Wiener diversity index; **(B)** Simpson’s dominance index; **(C)** Margalef’s richness index; **(D)** Pielou’s equitability index, and **(E)** Evar species evenness index. Significant differences (*P*< 0.05) in the treatments are indicated by uppercase letters (A–C) for each year. For each treatment, there was no significant (ns) differences among the years.

Five species diversity indexes were used to analyze community species abundance: Margalef’s species richness index (D_ma_, Equation 2), the Shannon-Wiener diversity index (H, Equation 3), Simpson’s dominance index (C, Equation 4), the Evar species evenness index (E_var_), and Pielou’s evenness index (E_pi_, Equation 5). The results showed that nutrient addition had no pronounced effect on each index (**Table 2**, *P >* 0.05), but years significantly affected species diversity (**Table 2**, *P<* 0.001). **Figure 6** shows that all the diversity indexes fluctuated among years with the lowest values recorded in 2022, except for the E_var_. Furthermore, the results also suggested that there was no interaction between year and nutrient addition and that year and nutrients influenced all the diversity indexes independently (**Table 2**, *P >* 0.05).

**Table 2:** Summary of the linear mixed-effect model relating fixed factors (Y, T) for the Shannon–Wiener diversity index (H); Simpson’s dominance index (C); Margalef’s richness index (D_ma_); Pielou’s equitability index (E_pi_) and Evar species evenness index (E_var_).

Source	H		C		D_ma_		E_pi_		E_var_	
	*F*	*P*	*F*	*P*	*F*	*P*	*F*	*P*	*F*	*P*
Y	17.999	0.000***	18.680	0.000***	96.792	0.000***	15.176	0.000***	12.550	0.000***
T	0.658	0.623	1.108	0.359	0.717	0.583	0.664	0.619	0.668	0.616
Y×T	1.269	0.239	1.265	0.242	0.917	0.554	1.196	0.292	1.690	0.067

Y represents different years, T represents the different treatments, and Y × T is their interaction. ***Significant difference at the P< 0.05 level.

### 3.5 Relationship between biomass and biodiversity

**Figure 7:**
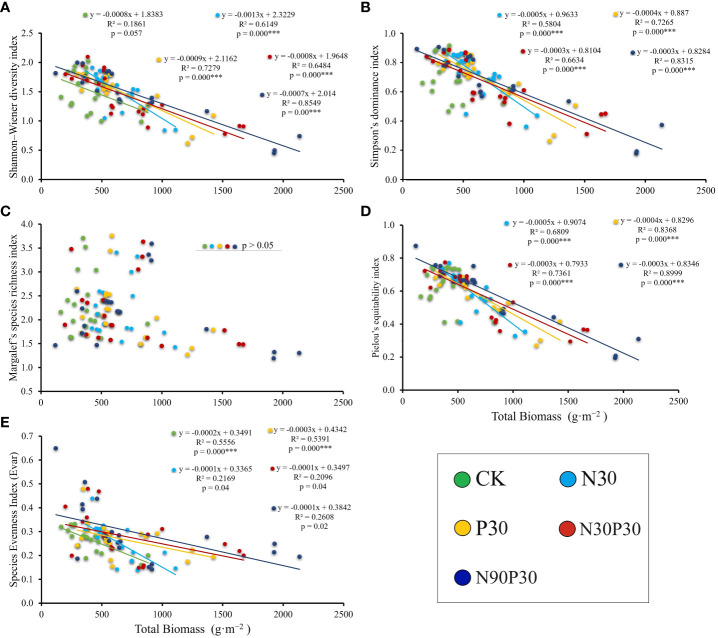
Relationships between biodiversity indexes and total above-ground biomass. **(A)** Shannon–Wiener diversity index, **(B)** Simpson’s dominance index, **(C)** Margalef’s richness index, **(D)** Pielou’s equitability index, and **(E)** Evar species evenness index. ***Significant difference at the P< 0.05 level.

Separate analyses revealed various relationships between the total AGB and biodiversity (**Figure 7**). The total AGB was negatively correlated with the Shannon-Wiener biodiversity index over the 5 years *(P<* 0.05) and with the Simpson’s dominance index *(P<* 0.05) in N30, P30, N30P30, and N90P30, but CK was not affected by total AGB *(P >* 0.05). The Margalef’s species richness index was independent of the total AGB across all treatments (*P >* 0.05). The Pielou’s equitability index was negatively correlated with total AGB under all treatments (*P<* 0.05) except CK (*P* > 0.05). The total AGB was negatively correlated with the species evenness index (Evar) for all treatments (*P<* 0.05).

## 4 Discussion

### 4.1 Effect of nutrient addition on above-ground biomass

In a temperate grassland in China, Wang et al. (2019) found that total net primary production responses to N addition were driven by above-ground plant responses; in a cross-biome meta-analysis, Li et al. (2016) showed that fertilization with either N or P stimulated aboveground biomass; and Keller et al. (2023) found that fertilization strongly affected aboveground growth, with both N and P addition stimulating aboveground biomass in US grasslands. The addition of N and P to the experimental plots used in this study generally positively affected grassland AGB. However, N and P saturation was observed after the five-year experiment. These results agreed with previous studies on mountain meadows in Tajikistan (Ikramova and Madaminov, 1968; Madaminov, 1969; Madaminov, 1970). One of the hypotheses in this study was that N and P fertilizer application would improve biomass production. This hypothesis was verified because N addition significantly increased community production. The total AGB significantly increased in 2018 under the N30P30 and N90P30 fertilizer treatments compared to CK. Additionally, for the years 2021 and 2022, the total AGB and all the *P. pabularia* indexes greatly outperformed CK, especially in the N30P30 and N90P30 treatments. The results also implied that some species in the grassland were more sensitive to mineral fertilizer treatment than *P. pabularia*. It is possible that *P. pabularia* may have lower requirements for some nutrients because it is more adaptable to soil conditions and can fully absorb the required nutrients from the soil, but further research is needed to confirm this. Total AGB and PB were not significantly different in 2019 and the reason could the low temperature (6.77° C, **Figure 1A**) and high precipitation (1211.90 mm, **Figure 1B**) in this year.

These results confirm that plants, especially the dominant population, have a number of responses to nutrient addition in Tajikistan mountain meadows, which is consistent with findings for the Songnen grassland of China (Gao et al., 2019). Across all treatment combinations over five years, the mean effect on total AGB was +12,51% for CK, +19.19% for N30, +20.7% for P30, +22.47% for N30P30, and +25.13% for N90P30, indicating that N30P30 and N90P30 increased biomass the most. The mean effect on PB over five years across all treatments was +10.65% for CK, +17.92% for N30, +20.18% for P30, +23.27% for N30P30, and +27.97% for N90P30, indicating that *P. pabularia* responded in the same way to total AGB. The results from this study show that a moderate application of mineral fertilizer is the optimum choice for increasing the aboveground biomass of *P. pabularia*.

### 4.2 Influence of nutrition addiction on biodiversity

Community species diversity is reflected by the Margalef index. It is common practice to compare species diversity using this index because it can determine a large number of species and the degree of their abundance in a community (Poole and Morgan, 1974; Vance-Chalcraft et al., 2010). The Pielou’s equitability index, Simpson’s dominance index, Evar species evenness index, and Shannon-Wiener diversity index are also common indices of biodiversity. Species variety may improve yield stability, according to the ecological literature (Haughey et al., 2018; Bocci et al., 2020). The results from this study showed that there were significant variations in the Margalef index and the other indexes for the various treatments over the five-year study period. In comparison to the first year (2018), the richness index for the mineral fertilizers decreased in the fourth and fifth years (2021 and 2022). An earlier study revealed that N addition decreased the compositional stability of grasslands, which could be due to an increase in N availability (Xu et al., 2012), and Lai et al. (2018) demonstrated that N addition significantly decreased understory plant diversity, especially in high-N plots. The Margalef index, Shannon-Weiner index, Simpson dominance index, and Pielou equitability index indicated that species richness dropped over the five years, but the Evar species evenness index significantly increased. The results from this study were similar to those reported by other studies (Strengbom et al., 2002; Gilliam, 2006). The fundamental factor driving the reduction in species variety in mountain meadows is competition. Bobbink et al. (2010) found that N deposition strongly reduced plant diversity in temperate and northern parts of North America and Europe because N deposition is a major driver of species composition change. Species that cannot adapt to their environment become extinct as a result of competitive exclusion, which eventually results in decreased variety. The N addition rates in this study were high and may have surpassed the N demand for plants and soil microbes. This can induce soil acidification and toxic ions release (Chen et al., 2013), which reduces plant diversity. For example, McClean et al. (2011) studied N deposition in Europe from the 1950s to the 1990s and discovered that environmental variables, such as N deposition and land use, considerably reduced variety by causing local plant extinctions and by affecting plant competitiveness. The results from this study showed that mineral fertilizers led to a lower richness index in the fourth and fifth years (2021 and 2022) compared to the first year (2018). This could be due to the increased nitrogen (N) supply as N addition has been shown to reduce the compositional stability of grasslands (Liu et al., 2019).

The IV represents species function and its status in the plant community and it may also indicate whether a species has dominant position in the community (Wenye et al., 2010; Asigbaase et al., 2019). *Prangos pabularia* is a well-known dominant species in the grasslands of the Ziddi region in northwest Tajikistan. According to this study, the *P. pabularia* IV for mineral treatments was higher in 2021 and 2022 than it was in 2018 (**Figure 3**). The proportional biomass analysis of the various species in the community (**Figure 4**) further suggested that mineral fertilizer could enhance not only *P. pabularia* yields but also the function of the dominant species in the community. This may be related to the fact that *P. pabularia* can more efficiently uptake and utilize N than other species. However, the grassland in this region is cut by farmers who require fodder grass to feed animals. Consequently, there is considerable interest in increasing *P. pabularia* productivity. Based on the intended purpose of the grassland, researchers should pay special attention to fertilization and take appropriate action.

### 4.3 Impact of nutritional variables on the biomass-biodiversity relationship

The relationship between above‐ground grassland biomass and diversity is an important ecological topic (Fayiah et al., 2019; Li et al., 2019) as grasslands are an important resource for local livestock development. However, previous studies have revealed different biomass–diversity relationships around the world, such as positive linear relationships, negative linear relationships, and nonlinear relationships (Bai et al., 2007; Grace et al., 2016; Zhang et al., 2022; Ye et al., 2023). In this study, all the diversity indexes had a significant negative correlation with total AGB except for Margalef species richness (*P<* 0.05) (**Figure 7**). The major differences between the negative biomass-diversity results obtained in this study and the other widely detected relationships could be linked to nutrient levels and light competition (Zhou et al., 2016; Li et al., 2017; Fayiah et al., 2019). In mountainous areas, climate factors also regulate plant growth (Fan et al., 2021). In this study, the AGB of the dominant species fluctuated among the years with the highest values occurring in 2022 when the diversity index values were lowest (**Figures 2**, **6**). This may have been because *P. pabularia* occupied more habitat space and resources in the well-resourced environments, leading to an increase in its biomass and biomass suppression in other species, which would reduce diversity. Combining the diversity analysis with precipitation and temperature showed that precipitation and temperature in 2022 were suitable for plant growth (Fan et al., 2021; **Figures 1**, **2**). As a result, an increase in the AGB of the dominant species decreased species diversity (Stevens et al., 2004; Koerner et al., 2018) through light competition (Hautier et al., 2009; DeMalach and Kadmon, 2017), which caused a negative biomass-diversity relationship. This negative correlation suggests that the habitat needs of species needs to be balanced with resource use when attempting to conserve and manage ecosystems to maintain ecosystem stability and diversity.

Apart from light competition, nutrient addition also leads to a negative biomass-diversity relationship. After fertilization, the increase in AGB of the dominant species in the study area was accompanied by an increase in the height of the dominant species (**Supplementary Table 1**) compared to the other species, which may have limited the growth of other species through strong competition for nutrients and light (Koerner et al., 2018). This would enhance the negative correlation between biomass and diversity, which was particularly prominent in the lower nutrient addition treatment. However, this kind of negative relationship was alleviated in the higher nutrient addition treatments where the added nutrients compensated for resource competition among species.

In general, the results showed that adding mineral fertilizers may rapidly increase *P. pabularia* AGB in mountain meadows (Tong et al., 2019). Overall, *P. pabularia* was the prime determinant of community productivity and the diversity responses to N and P addition.

## 5 Conclusions

*Prangos pabularia* is an excellent forage grass and was the dominant species in the study area. Mineral fertilizers increased the *P. pabularia* proportion in the plant community and enhanced the establishment of the dominant species in the Ziddi grassland, which should lead to improvements in forage quantity and quality in the region. The hypotheses were that N and P fertilizer application would increase plant AGB and perhaps community diversity. The results showed that the combined application of N and P fertilizers over a five-year period enhanced total AGB as well as *P. pabularia* AGB and height. However, the increasing impact of the dominant species is not advantageous for species diversity, especially when nutrients are added, which may exacerbate species loss through nutrient and light competition. In summary, grassland has the ability to recover on its own, albeit at a slow pace. Mineral fertilizers increased the *P. pabularia* proportion in the plant community and enhanced the establishment of dominant species in the Ziddi grassland, but they may also reduce grassland biodiversity. Therefore, mineral fertilizer utilization should be based on the purpose of the grassland, such as producing fodder, grazing, or ecological improvement.

## Data availability statement

The original contributions presented in the study are included in the article/**Supplementary Material**. Further inquiries can be directed to the corresponding author.

## Author contributions

All the authors showed in the article contributed to study conceptualization. Y-ML and LF conceived the study. OM conducted the experiments, analyzed the data, and drafted the manuscript. MA and YS performed the experiments. All authors contributed to the article and approved the submitted version.
