# Supplementary material for: Nutrient addition alters plant community productivity but not the species diversity of a mountain meadow in Tajikistan
Source: Front Plant Sci. 2024 Jan 15;14:1235388. doi: 10.3389/fpls.2023.1235388 (PMC10822985; doi:10.3389/fpls.2023.1235388)
Supplement: Supplementary file 1 [file DataSheet_1.pdf]

Supplementary Material

Table S1. The effect of different treatments on *P. pabularia* height in each year (mean ± SE).

Year		CK	N30	P30	N30P30	N90P30
2018	Height (cm)	200.00±7.07a	220.00±9.13a	230.00±5.77a	220.00±20.82a	200.00±8.66a
2019	Height (cm)	190.00±4.08a	207.50±4.79a	220.00±10.00a	226.67±8.82a	211.67±6.01a
2020	Height (cm)	165.75±10.71b	217.50±5.95a	210.00±7.07a	226.67±14.53a	227.50±10.44a
2021	Height (cm)	110.00±5.00b	116.50±1.94b	117.67±1.45b	167.50±17.50b	150.00±0.00b
2022	Height (cm)	95.00±10.00b	93.50±8.50b	108.33±8.11a	116.67±4.41b	94.67±5.04b

Note: Different letters in the same rows indicate a significant difference at the 0.05 level (P<0.05*).*

Table S2. Total aboveground biomass and *P. pabularia* biomass in different treatments ($g \cdot m^{-2}$, mean ± SE).

Year		CK	N3	P3	N3P3	N9P3
2018	Total	450.70±38.00b	756.90±50.00ab	613.70±33.10ab	839.90±27.10a	919.20±23.40a
	Prangos	180.60±14.73b	422.30±25.08ab	283.20±6.74b	576.40±15.48a	584.40±5.16a
2019	Total	406.30±17.20ab	501.70±24.70a	537.80±17.60a	549.30±42.20a	598.30±35.60a
	Prangos	78.10±6.64b	159.03±8.79ab	192.45±4.15ab	193.34±13.89ab	241.24±11.32a
2020	Total	527.22±23.79ab	545.40±26.34ab	582.31±19.08a	605.49±21.09a	672.30±40.09a
	Prangos	283.00±7.60ab	239.75±6.18ab	305.40±7.25ab	392.00±4.73a	427.50±10.44a
2021	Total	559.11±53.42b	835.00±32.03ab	929.58±36.31ab	1133.08±61.37a	1400.58±18.44a
	Prangos	321.50±34.50b	422.75±8.51b	564.00±16.92ab	680.00±32.00ab	966.00±00a
2022	Total	897.00±23.28b	1316.83±23.38ab	1481.00±37.25ab	1792.00±36.37a	2252.50±41.13a
	Prangos	573.00±11.00b	841.00±6.00b	1051.33±9.39ab	1266.33±11.57ab	1745.67±14.08a

Note: Different letters in the same rows indicate a significant difference at the 0.05 level (P<0.05*).*

Table S3. Summary of the linear mixed-effect model relating fixed factor (Y, T) for *P. pabularia*, *G. colinum*, *V. tenuifolia*, *A. corydalinus*, *C.sibirica*, and other plants.

SOURCE	Prangos		Geranium		Vicia		Astragalus		Crepis		Others	
	F	P	F	P	F	P	F	P	F	P	F	P
**Y**	1.54E+31	0.000*	1.42E+30	0.000*	3.60E+29	0.000*	7.29E+29	0.000*	3.45E+29	0.000*	4.76E+29	0.000*
**T**	4.79E+30	0.000*	3.25E+29	0.000*	1.26E+30	0.000*	1.16E+29	0.000*	2.08E+28	0.000*	2.97E+29	0.000*
**Y×T**	6.88E+29	0.000*	9.99E+28	0.000*	4.97E+29	0.000*	2.78E+29	0.000*	7.47E+28	0.000*	1.67E+29	0.000*

*Y means different years and T means different treatments, Y × T means the interaction. ***Significant difference at P < 0.05 level.*

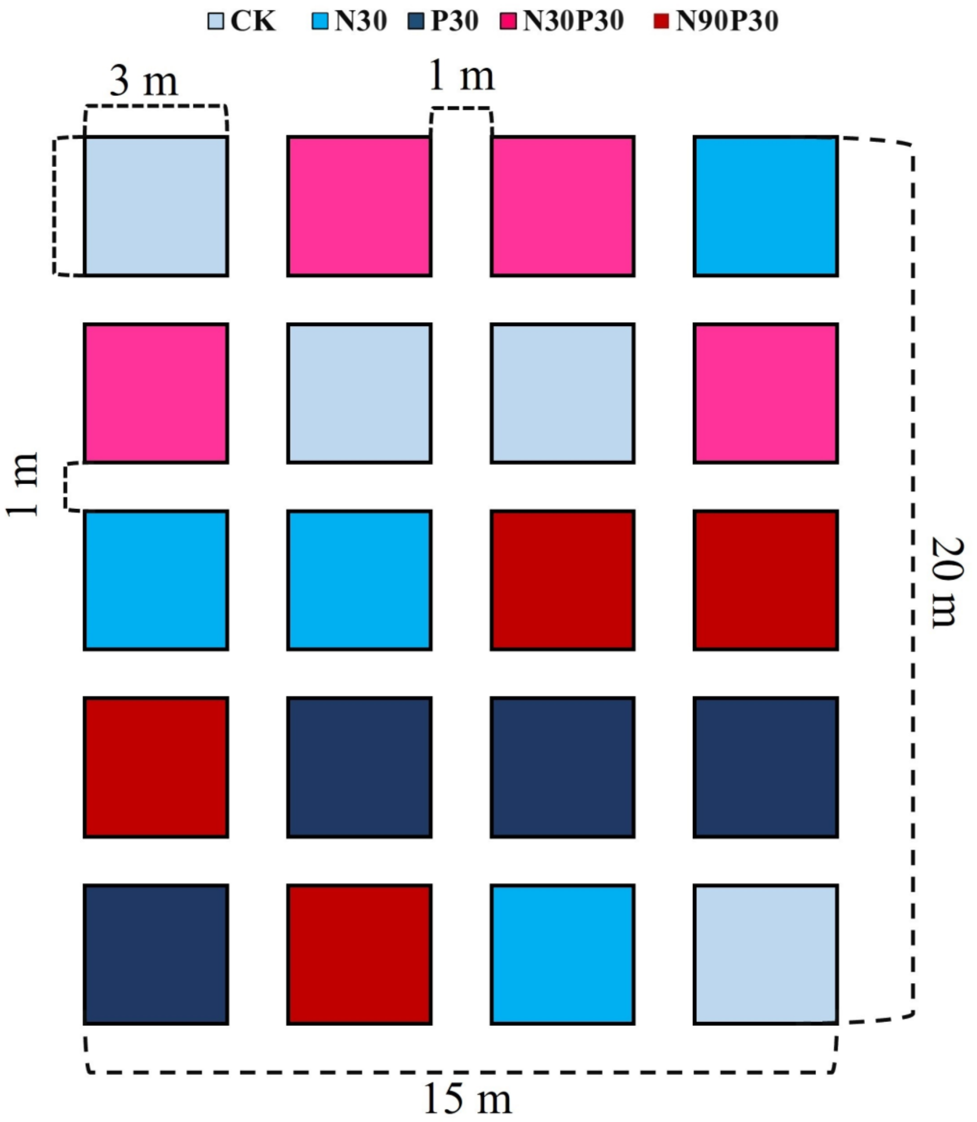

Figure S1. The experimental design. CK–the control; N30 - adding 30 kg N ha^{-1} $year^{-1}$, P30 - adding 30 kg P ha^{-1} $year^{-1}$, N30P30 - adding 30 kg N h a^{-1} $year^{-1}$ and 30 kg P ha^{-1} $year^{-1}$, N90P30- adding 90 kg N ha^{-1} $year^{-1}$ and 30 kg P ha^{-1} $year^{-1}$.
